# Association between short birth intervals and childhood anemia severity in India: evidence from the fifth National Family Health Survey (NFHS-5)

**DOI:** 10.3389/fnut.2026.1945328

**Published:** 2026-10-23

**Authors:** Soumen Barik, Arpita Show, Manoj Sonune

**Affiliations:** 1 Department of Fertility and Social Demography, International Institute for Population Sciences (IIPS), Mumbai, Maharashtra, India; 2 Department of Geography, Sadhu Ram Chand Murmu University of Jhargram, Jhargram, West Bengal, India; 3 School of Health Systems Studies, Tata Institute of Social Sciences, Mumbai, Maharashtra, India

**Keywords:** childhood anemia, health inequities, India, maternal nutritional depletion, NFHS-5, short birth interval

## Abstract

**Background:**

Anemia affects 67.1% of Indian children aged 6–59 months. Short birth intervals (SBI) may exacerbate this burden through maternal nutritional depletion; however, empirical evidence linking SBI with specific levels of childhood anemia severity remains limited in India.

**Methods:**

We analyzed nationally representative, secondary cross-sectional data from the National Family Health Survey-5 (NFHS-5, 2019–21), comprising an unweighted analytical sample of 101,240 mother–child pairs with children aged 6–59 months. Survey-adjusted multinomial logistic regression was used to assess the association between SBI and anemia severity (mild, moderate, and severe). Adjusted odds ratios (aOR) were estimated after controlling for key child, maternal, household, and geographic characteristics.

**Results:**

Compared with non-short birth interval (NSBI), SBI was significantly associated with a higher odd of moderate anemia (aOR: 1.10; 95% CI: 1.05–1.15) and severe anemia (aOR: 1.18; 95% CI: 1.05–1.34), but not mild anemia. The highest burden of severe anemia was observed among children aged 6–23 months, those with birth weight <2,500 g, and those born to mothers with severe anemia. Children from Scheduled Caste/Scheduled Tribe (SC/ST) communities, and the West, North, Central and South regions had higher odds of severe anemia, whereas higher maternal education, greater household wealth, and residence in the Northeast were associated with a lower odd of anemia.

**Conclusion:**

SBI is associated with a higher odd of moderate and severe anemia among children in India, potentially associated with maternal nutritional depletion and intersecting structural inequities. Addressing this public health burden requires integrated strategies that promote optimal birth spacing, strengthen family planning services, and provide targeted maternal nutrition interventions for marginalized populations, thereby supporting progress toward the Sustainable Development Goals.

## Introduction

Anemia is a blood disorder characterized by a reduced concentration of hemoglobin or a decreased number of red blood cells (RBCs), resulting in impaired oxygen-carrying capacity and an inability to efficiently transport oxygen throughout the body (1, 2). Globally, an estimated 40% of children aged 6–59 months were affected by anemia in 2019. The burden was particularly high in South Asia, where 52% of children in this age group were anemic, accounting for nearly 87 million cases (3). According to NFHS-5 approximately two-thirds (67.1%) of Indian children aged 6–59 months are anemic, making it one of the country’s most pressing child nutrition and public health challenges (4). While iron deficiency is the primary cause, deficiencies in other essential micronutrients (e.g., vitamins A, B6, B12, folate) and chronic infections also contribute to its development (5). If left untreated during early childhood, anemia leads to impaired psychomotor, cognitive, neurological, and socioemotional development, with potentially long-lasting consequences for learning and behavior (6, 7) Furthermore, it increases susceptibility to bacterial and gastrointestinal infections and elevates the risk of child mortality (8–10). While previous studies have identified various child, maternal, and household determinants of childhood anemia disparities (11–18).

Short birth interval (SBI), typically defined as less than 24 months between a live birth and the next conception (or less than 33 months between successive live births), is a well-established determinant of adverse maternal, perinatal, and child health outcomes globally (18–20). In India, nearly half of women (47.8%) experienced an SBI during 2019–21, reflecting ongoing challenges in family planning adoption and highlighting its potential implications for adverse child health outcomes (21). Global and regional evidence consistently links SBI to increased risks of preterm birth, low birth weight, neonatal mortality, and childhood undernutrition (20, 22, 23). However, its specific role in exacerbating the burden of childhood anemia requires deeper investigation.

The association between SBI and childhood anemia is primarily explained by the maternal nutritional depletion hypothesis. Pregnancy and lactation substantially deplete maternal iron and other essential micronutrient stores, including folate, vitamins A and B12, zinc, and calcium (20, 24, 25). Closely spaced pregnancies limit the time needed to replenish these nutrients, increasing the risk of maternal anemia and poor nutritional status (24, 26, 27). Biologically, poor maternal nutrition limits the transfer of iron to the fetus, resulting in lower neonatal iron stores and increasing the risk of low birth weight and preterm birth, both of which are strongly associated with early-onset childhood anemia (28–30). Furthermore, maternal micronutrient deficiencies may compromise breast milk quality, depriving the nursing infant of essential bioavailable iron (22, 31, 32). Socially, closely spaced births can dilute household dietary, healthcare, and financial resources among multiple young children, while concurrent childcare and pregnancy may lead to early weaning and delayed introduction of nutrient-rich complementary foods (23, 33, 34). Together, these factors create a cumulative risk environment that increases the severity of childhood anemia (35).

To address these interconnected challenges, the Government of India has implemented various initiatives, including the National Family Planning Programme to promote optimal birth spacing, and nutrition-specific interventions such as the POSHAN Abhiyaan and Anemia Mukt Bharat, which focus on maternal and child micronutrient supplementation. Despite these efforts, the high prevalence of both SBI and childhood anemia persists, indicating a need to better understand their intersecting pathways. Therefore, the primary objective of this study is to examine the association between SBI and childhood anemia severity using nationally representative data from India. Despite plausible biological and behavioral pathways linking SBI to childhood anemia, empirical evidence on this association particularly across different graded levels of anemia severity (mild, moderate, and severe) remains limited in the Indian context. Most existing studies treat anemia as a binary outcome or focus on general nutritional status, overlooking the distinct factors that may drive progression to moderate or severe anemia. Addressing this critical gap, the present study examines the relationship between SBI and specific levels of anemia severity among children aged 6–59 months. The findings will contribute to evidence-based, targeted interventions aimed at improving maternal and child health, optimizing birth spacing, and accelerating progress toward the Sustainable Development Goals (SDGs), particularly SDG 2 (Zero Hunger) and SDG 3 (Good Health and Well-being).

## Data methods

This study utilized secondary cross-sectional data from the fifth round of the National Family Health Survey (NFHS-5, 2019–2021), a nationally representative household survey conducted across India. The survey was implemented by the International Institute for Population Sciences (IIPS), Mumbai, under the stewardship of the Ministry of Health and Family Welfare (MoHFW), Government of India, with technical support from the Demographic and Health Surveys (DHS) Program and financial support from partners including USAID, UNICEF, UNFPA, the Bill & Melinda Gates Foundation, and the MoHFW.

NFHS-5 provides comprehensive, standardized information on population health, maternal and child nutrition, fertility, and healthcare utilization. Using a multistage stratified sampling design, the survey successfully covered 636,699 households, 724,115 women aged 15–49 years, and 232,920 children aged 0–59 months across all states and union territories of India. Data were collected using standardized questionnaires administered through computer-assisted personal interviewing (CAPI) in 18 Indian languages. Rigorous quality assurance procedures including extensive fieldworker training, close field supervision, and respondent back-checks ensured the reliability and validity of the data, making the NFHS-5 a robust and appropriate data source for examining the association between SBI and childhood anemia severity in India.

## Statistical analysis

### Phase 1: descriptive and bivariate analysis

Unweighted frequencies and survey-weighted percentages were used to describe the sample characteristics and the distribution of anemia severity. Bivariate associations between categorical independent variables and anemia severity were assessed using the survey design-adjusted Rao–Scott chi-square test. This test applies a design-effect correction to the standard Pearson chi-square statistic, appropriately accounting for the complex survey design by incorporating sampling weights, primary sampling units (clusters), and strata. All bivariate and multivariate survey analyses were conducted using the svy suite of commands in Stata. A two-sided *p*-value < 0.05 was considered statistically significant.

### Phase 2: multinomial logistic regression

Although childhood anemia severity is ordinal (no anemia, mild, moderate, and severe), the proportional-odds assumption was assessed using the Brant test and was violated for the overall model (*χ*^2^ = 463.07, *p* < 0.001), including for SBI (*χ*^2^ = 12.09, *p* = 0.002) and several covariates. This indicates that the effects of the predictors differ across anemia severity thresholds. Given this violation, multinomial logistic regression was considered more appropriate for estimating category-specific associations.

A survey-weighted multinomial logistic regression model was employed to examine the association between SBI and anemia severity among children aged 6–59 months. The outcome comprised four categories: no anemia, mild, moderate, and severe anemia, with no anemia specified as the reference category. Non-short birth interval (NSBI) and the first category of each covariate were specified as reference groups. Adjusted odds ratios (aOR) with 95% confidence intervals (CIs) were estimated. The NFHS-5 complex survey design was accounted for by incorporating the sampling weight (v005), primary sampling unit (v021), and stratification variable (v022) using svyset, with linearized variance estimation and single-unit strata centered at the overall mean. This approach ensures that the estimated standard errors and confidence intervals account for the complex survey design (36).

The multinomial logistic regression model is expressed as:

$$\ln(\frac{P(Y_{i}=j)}{P(Y_{i}=0)})=\beta_{0j}+\beta_{1j}X_{1i}+\beta_{2j}X_{2i}+\cdots +\beta_{\mathrm{pj}}X_{\mathrm{pi}},j=1,2,3$$

where $Y_{i}$ denotes the anemia status of child i , j represents the categories of mild, moderate, and severe anemia, and $P(Y_{i}=0)$ denotes the probability of being not anemic (reference category).

The exponentiated coefficients, e^(β_j), are interpreted as adjusted odds ratios (aORs). An aOR greater than 1 indicates higher odds of being in a given anemia category relative to the reference category (not anemic), whereas an aOR less than 1 indicates lower odds. Statistical significance was assessed at *p < 0.10, **p < 0.05, and ***p < 0.01. Mathematically, the multinomial logit model estimates the log-odds of the j*j*-th outcome relative to the base category $\ln(P_{j}/P_{k})=X$ , meaning the exponentiated coefficients strictly represent conditional odds ratios (CORs). Although statistical software defaults label these as relative risk ratios (RRRs), we report them as adjusted odds ratios (aORs) to provide the statistically exact interpretation of covariate effects on the relative odds of anemia severity versus the baseline, independent of the probabilities of the remaining outcome categories. Potential multicollinearity among covariates was assessed using variance inflation factors (VIFs). The mean VIF was 1.62, with all VIFs below 3.1, indicating no substantial multicollinearity among the covariates included in the fully adjusted model. All statistical analyses were conducted using Stata (Version 17.0; StataCorp LLC, College Station, TX, United States), accounting for the complex survey design. Data management and preparation were also performed in Stata, while all figures and flowcharts were generated using R software (Version 4.3.1; R Foundation for Statistical Computing, Vienna, Austria) using the ggplot2 package.

### Outcome variable

The primary outcome variable was childhood anemia severity among children aged 6–59 months, obtained from the NFHS-5 variable (*hw57*). Hemoglobin concentration was measured using a battery-operated portable HemoCue Hb 201 + analyzer, and values were adjusted for altitude following NFHS-5 protocols.

Based on the World Health Organization (WHO) and NFHS-5 guidelines, anemia status was classified into four categories:

- 0 = Not anemic: hemoglobin ≥ 11.0 g/dL
- 1 = Mild anemia: hemoglobin 10.0–10.9 g/dL
- 2 = Moderate anemia: hemoglobin 7.0–9.9 g/dL
- 3 = Severe anemia: hemoglobin < 7.0 g/dL

After applying the study’s exclusion criteria, the final analytical sample comprised 101,240 children aged 6–59 months with valid hemoglobin measurements and complete information on all study variables.

### Predictor variable

Birth interval was defined as the number of months between the index child’s birth and the immediately preceding live birth. Following World Health Organization (WHO) and Demographic and Health Surveys (DHS) guidelines, a short birth interval (SBI) was defined as an interval of <33 months between two consecutive live births, whereas intervals of ≥33 months were classified as NSBI and used as the reference category. The preceding birth interval was obtained from the NFHS-5 variable b11. Children without a preceding live birth, including first-born children, were not assigned a birth-interval category and were excluded from analyses involving birth interval. For mothers with three or more births, each eligible child born within the 5 years preceding the survey was assigned the birth interval preceding that child’s birth; consequently, more than one eligible child could contribute to the analysis from the same mother. For the purpose of analysis, SBI was dichotomously coded as 1 if the preceding birth interval was less than 33 months, and 0 otherwise (21, 37). Covariates were selected *a priori* based on their theoretical relevance, evidence from previous literature, and availability in the NFHS-5 dataset (21, 23, 38, 39). The operational definitions and classifications of all study variables included in the analysis are detailed in Table 1.

**Table 1:** Operational definitions and classification of study variables included in the analysis.

Variable	Response description
Child characteristics	
Child sex	Male, female
Child age (months)	The age of the child was grouped into three categories: 6–23 months, 24–41 months, and 42–59 months.
Birth weight	Birth weight was categorized into three groups: (≥2,500 g), (<2,500 g), and not weighed.
Birth order	Birth order was categorized into two groups: second birth and third or higher birth.
Maternal characteristics	
Maternal age (years)	Women’s age was categorized into three groups: 15–24 years, 25–34 years, and ≥35 years.
Maternal education	Maternal education was categorized into four groups: no education, primary education, secondary education, and higher education.
Age at first birth	Age at first birth was categorized into three groups: <20 years, 20–29 years, and ≥30 years.
Place of delivery	Place of delivery was classified as non-institutional and institutional delivery.
Maternal anemia	Maternal anemia was classified as not anemic, mild, moderate, and severe based on hemoglobin concentration (Hb ≥ 12.0, 10.0–11.9, 7.0–9.9, and <7.0 g/dL, respectively).
Maternal Body Mass Index (BMI)	Maternal BMI was categorized as underweight (<18.5 kg/m^2^), normal (18.5–24.9 kg/m^2^), and overweight/obese (≥25.0 kg/m^2^).
Received any benefits from the Anganwadi	Receipt of anganwadi benefits was classified as yes or no based on whether the respondent received any services or benefits from an anganwadi Centre.
Exposure to mass media	Mass media exposure was classified as any exposure or no exposure based on respondents’ exposure to newspapers/magazines, radio, television, or cinema/theatre.
Household and community factors	
Wealth index	The wealth index was categorized into five quintiles: poorest, poorer, middle, richer, and richest, based on the NFHS-5 household wealth index derived using principal component analysis (PCA).
Household members	Household size was categorized into three groups: 1–4 members, 5–8 members, and more than 8 members.
Social caste	Social caste was categorized into three groups: Other (General and other castes), Other Backward Classes (OBC), and Scheduled Castes/Scheduled Tribes (SC/ST).
Religion	Religion was categorized into three groups: Hindu, Muslim, and other religions.
Residents	Place of residence was categorized into two groups: urban and rural.
Geographical region	Geographical region was categorized into six regions following the NFHS classification: *Northern* (Jammu & Kashmir, Ladakh, Himachal Pradesh, Punjab, Rajasthan, Haryana, Uttarakhand, Chandigarh, and Delhi); *Central* (Uttar Pradesh, Madhya Pradesh, and Chhattisgarh); *Eastern* (Bihar, Jharkhand, West Bengal, and Odisha); *Western* (Gujarat, Maharashtra, Goa, and Dadra & Nagar Haveli and Daman & Diu); *Southern* (Kerala, Karnataka, Andhra Pradesh, Tamil Nadu, Andaman & Nicobar Islands, Puducherry, and Lakshadweep); and *Northeastern* (Sikkim, Assam, Meghalaya, Manipur, Mizoram, Nagaland, Tripura, and Arunachal Pradesh).

Figure 1 presents the flowchart of the sample selection process. The study initially included 232,920 mother–child pairs from the NFHS-5 Kids Recode (KR) dataset, comprising women aged 15–49 years. Never-married women (*n* = 334) were excluded because, in the Indian context, childbearing predominantly occurs within marriage, and the study focused on birth intervals within established marital unions. This resulted in 232,586 ever-married women. Subsequently, 59,460 mothers with no children or only one child were excluded, as birth interval calculation requires at least two live births, resulting in 173,126 mothers having at least two children.

**Figure 1:**
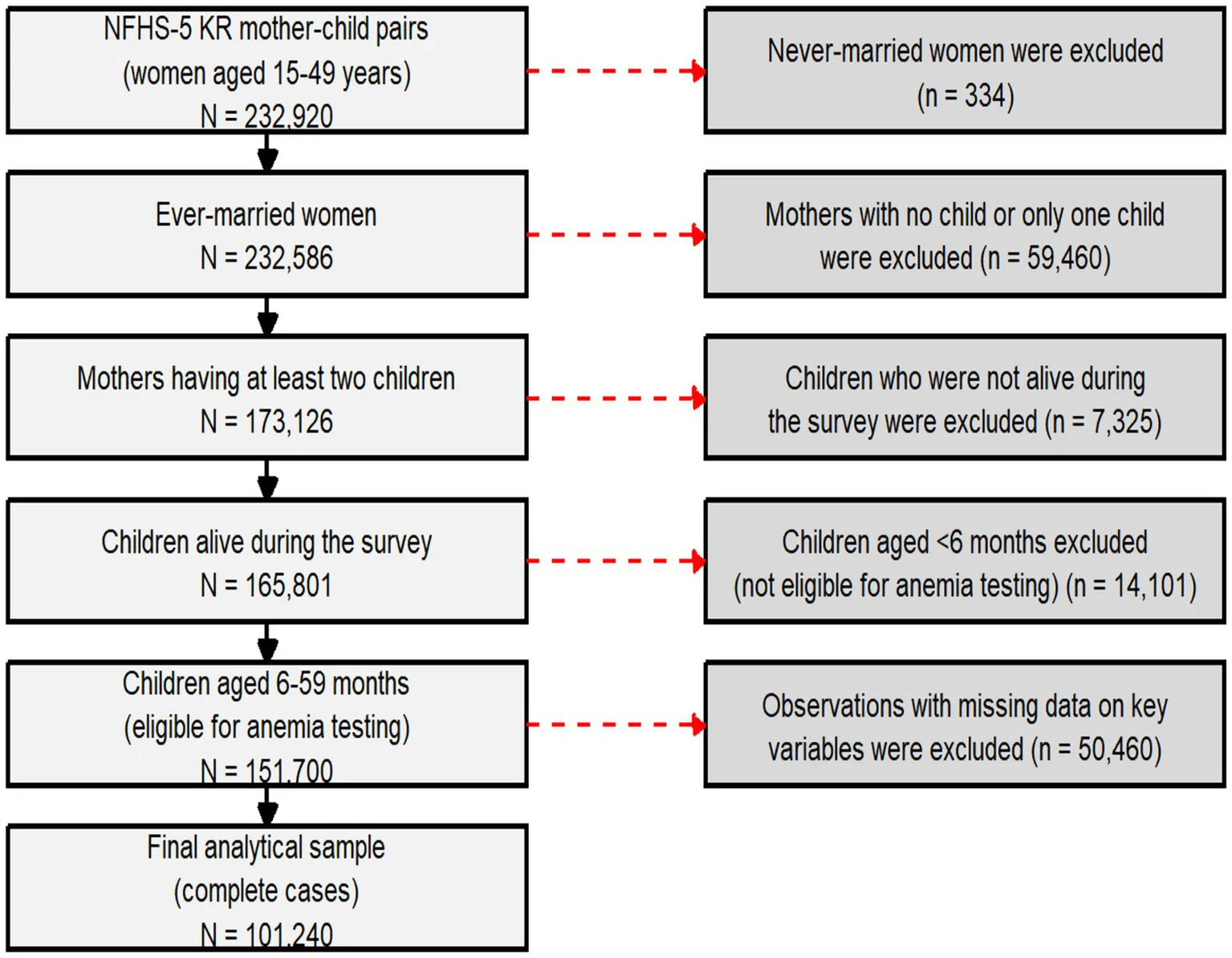
Flowchart of the sample selection process.

Further exclusions were made for 7,325 children who were not alive during the survey period, yielding 165,801 children alive during the survey. Finally, 14,101 children aged less than 6 months were excluded because they were not eligible for anemia testing according to NFHS-5 protocols Finally, 50,460 observations with missing data on key study variables, including childhood anemia severity (*n* = 12,895), birth interval (*n* = 25,036), maternal anemia (*n* = 1,032), maternal BMI (*n* = 6,603), and social category (*n* = 4,894), were excluded. These missing values primarily reflect non-response, unavailable measurements, or incomplete information recorded during the original NFHS-5 data collection. No imputation was performed; observations with missing values on the variables required for the analysis were excluded using a complete-case approach. After applying all exclusion criteria, 151,700 children constituted the eligible sample prior to missing-data exclusion. After excluding 50,460 children with missing data, the final analytical sample comprised 101,240 children aged 6–59 months and their mothers. Which was used to examine the association between short birth intervals (SBI) and childhood anemia severity in India.

Figure 2 illustrates the distribution of childhood anemia severity among children aged 6 to 59 months, stratified by mother birth interval status. Children born after a SBI exhibited a higher overall anemia burden compared to those born after a NSBI. Specifically, the proportion of non-anemic children was lower in the SBI group (29.9%) than in the NSBI group (32.2%). Conversely, SBI was associated with a shift toward greater anemia severity, demonstrating a higher prevalence of moderate (39.2% vs. 36.5%) and severe (2.3% vs. 2.1%) anemia compared to NSBI. The prevalence of mild anemia remained comparable between both groups (28.6% vs. 29.2%). Overall, these descriptive findings indicate an association between SBI and higher prevalence of moderate and severe childhood anemia.

**Figure 2:**
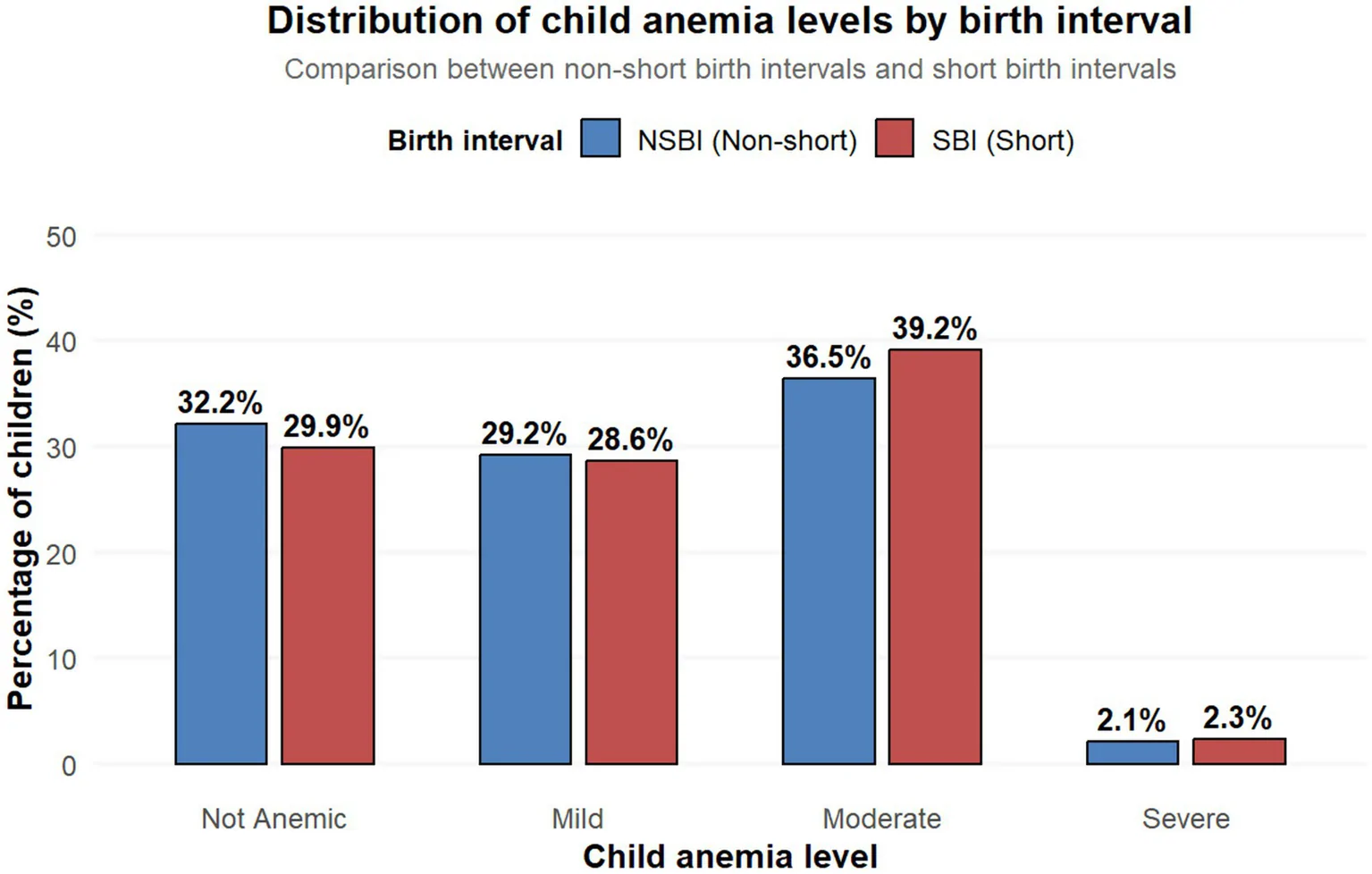
Distribution of childhood anemia severity by birth interval status.

The analytical sample Table 2 comprised 101,240 children aged 6–59 months, predominantly male (52.98%) and evenly distributed across age strata (6–23 months: 32.04%; 24–41 months: 33.91%; 42–59 months: 34.05%). Most children had a normal birth weight (74.49%) and were of second (55.16%) or higher birth order (44.84%). The majority of mothers were aged 25–34 years (70.0%) and delivered institutionally (85.64%). Maternal anemia was highly prevalent, with over 60% of women affected (mild: 26.33%; moderate: 31.69%; severe: 2.55%). Nearly half of the mothers had completed secondary education (47.14%), while 27.59% had no formal schooling. The sample was predominantly rural (75.31%) and concentrated in the poorest (27.78%) and poorer (22.74%) wealth quintiles. Socially, the cohort included substantial OBC (45.56%) and SC/ST (35.91%) representation, with 81.18% identifying as Hindu. Geographically, the Central (29.37%) and East (26.70%) regions accounted for the largest shares, followed by the South (15.92%), North (13.40%), West (12.09%), and Northeast (2.52%).

**Table 2:** Background characteristic of the study sample.

Child characteristics	Response description	Frequency, *n* (%)
Child sex	Male	53,382 (52.98)
	Female	47,858 (47.02)
Child age (months)	6–23	32,257 (32.04)
	24–41	34,292 (33.91)
	42–59	34,691 (34.05)
Birth weight	≥2,500 g	75,369 (74.49)
	<2,500 g	14,158 (14.81)
	Not weighed	11,713 (10.69)
Birth order	Second birth	53,788 (55.16)
	Third or higher birth	47,452 (44.84)
Maternal characteristics		
Women age (years)	15–24	15,655 (17.17)
	25–34	70,288 (70)
	> = 35	15,297 (12.83)
Age at first birth	Less than 20	38,390 (39.6)
	20 to 29	60,628 (58.71)
	30 to 49	2,222 (1.7)
Women education	No education	28,208 (27.59)
	Primary	15,366 (14.57)
	Secondary	48,180 (47.14)
	Higher	9,486 (10.7)
Place of delivery	Non-Institutional	17,269 (14.36)
	Institutional	83,971 (85.64)
Maternal anemia	Not anemic	40,855 (39.43)
	Mild	26,233 (26.33)
	Moderate	31,510 (31.69)
	Severe	2,642 (2.55)
Maternal BMI	Normal	63,373 (60.49)
	Underweight	19,570 (20.22)
	Overweight/Obese	18,297 (19.29)
Received any benefits from the anganwadi	No	27,782 (28.02)
	Yes	73,458 (71.98)
Exposure to mass media	No	32,397 (31.48)
	Any	68,843 (68.52)
Household and community factors		
Wealth index	Poorest	30,909 (27.78)
	Poorer	24,587 (22.74)
	Middle	19,152 (19.44)
	Richer	15,408 (16.93)
	Richest	11,184 (13.1)
Household members	1 to 4	21,082 (20.87)
	5 to 8	63,863 (62.1)
	More than 8	16,295 (17.03)
Social caste	Other	16,328 (18.53)
	OBC	39,757 (45.56)
	SC/ST	45,155 (35.91)
Religion	Hindu	75,177 (81.18)
	Muslims	12,412 (14.61)
	Others	13,651 (4.211)
Residents	Urban	19,050 (24.69)
	Rural	82,190 (75.31)
Geographic region	East	20,822 (26.7)
	West	8,804 (12.09)
	North	18,053 (13.4)
	South	12,133 (15.92)
	Central	27,280 (29.37)
	Northeast	14,148 (2.523)

Frequencies are unweighted, and percentages are survey-weighted, accounting for sampling weights, PSUs, and strata.

Table 3 reveals significant disparities in childhood anemia severity across most background characteristics (all *p* < 0.001), except child sex (*p* = 0.334). Moderate and severe anemia burdens were highest among children aged 6–23 months (49.81 and 3.40%) and those with low birth weight <2,500 g (39.98 and 3.26%). Maternal factors showed clear gradients: children of severely anemic mothers had the highest rates of moderate (49.68%) and severe (6.25%) anemia, while higher maternal education was strongly protective (severe anemia: 1.79% vs. 2.43% for no education). Socioeconomic disadvantages further elevated odds, particularly among the poorest households (41.43% moderate, 2.07% severe), SC/ST communities (40.69% moderate, 2.62% severe), and rural residents (38.75% moderate, 2.20% severe). Geographically, severe anemia peaked in the North (3.23%) and West (2.85%), while the Northeast exhibited the lowest burden (0.73% severe).

**Table 3:** Bivariate associations of childhood anemia severity with background characteristics among children aged 6–59 months.

Child characteristics	Response description	No anemia	Mild anemia	Moderate anemia	Severe anemia	*P*-value
Child sex	Male	17,120 (30.92)	15,067 (28.8)	19,919 (37.93)	1,276 (2.36)	0.3337
	Female	15,563 (31.12)	13,593 (29.01)	17,695 (37.78)	1,007 (2.09)	
Child age (months)	6–23	6,902 (20.68)	8,280 (26.11)	15,954 (49.81)	1,121 (3.40)	<0.001
	24–41	10,734 (29.43)	9,936 (29.58)	12,821 (38.59)	801 (2.41)	
	42–59	15,047 (42.31)	10,444 (30.84)	8,839 (25.89)	361 (0.97)	
Birth weight	≥2,500 g	24,535 (31.52)	21,569 (29.17)	27,698 (37.29)	1,567 (2.02)	<0.001
	<2,500 g	4,179 (29.59)	3,757 (27.18)	5,745 (39.98)	477 (3.26)	
	Not weighed	3,969 (29.41)	3,334 (29.37)	4,171 (38.9)	239 (2.32)	
Birth order	Second birth	17,777 (32.3)	15,143 (28.85)	19,713 (36.77)	1,155 (2.08)	<0.001
	Third or higher birth	14,906 (29.42)	13,517 (28.95)	17,901 (39.2)	1,128 (2.43)	
Maternal characteristics						
Women age (years)	15–24	3,952 (24.97)	4,255 (27.42)	7,042 (45.06)	406 (2.55)	<0.001
	25–34	22,992 (31.75)	20,041 (29.13)	25,679 (36.91)	1,576 (2.22)	
	> = 35	5,739 (35.08)	4,364 (29.61)	4,893 (33.42)	301 (1.9)	
Age at first birth	Less than 20	11,976 (30.26)	11,074 (29.2)	14,504 (38.34)	836 (2.20)	<0.001
	20 to 29	19,864 (31.46)	17,011 (28.7)	22,352 (37.58)	1,401 (2.27)	
	30 to 49	843 (33.08)	575 (28.41)	758 (36.54)	46 (1.97)	
Women education	No education	7,872 (27.39)	8,200 (29.46)	11,430 (40.73)	706 (2.43)	<0.001
	Primary	4,933 (29.69)	4,333 (28.82)	5,758 (39.2)	342 (2.3)	
	Secondary	16,228 (31.97)	13,542 (28.59)	17,360 (37.24)	1,050 (2.21)	
	Higher	3,650 (37.92)	2,585 (28.89)	3,066 (31.4)	185 (1.79)	
Place of delivery	Non-Institutional	5,949 (29.76)	4,891 (29.39)	6,128 (38.94)	301 (1.92)	0.005
	Institutional	26,734 (31.22)	23,769 (28.81)	31,486 (37.68)	1982 (2.29)	
Maternal anemia	Not anemic	16,461 (38.37)	11,428 (28.62)	12,342 (31.38)	624 (1.63)	<0.001
	Mild	8,033 (29.8)	7,753 (29.96)	9,919 (38.22)	528 (2.02)	
	Moderate	7,647 (23.64)	8,877 (28.84)	14,056 (44.67)	930 (2.84)	
	Severe	542 (21.33)	602 (22.74)	1,297 (49.68)	201 (6.25)	
Maternal BMI	Normal	20,624 (30.78)	18,154 (29.18)	23,269 (37.88)	1,326 (2.16)	<0.001
	Underweight	5,148 (25.88)	5,449 (28.22)	8,422 (43.21)	551 (2.70)	
	Overweight/Obese	6,911 (37.1)	5,057 (28.71)	5,923 (32.2)	406 (1.99)	
Received any benefits from the anganwadi	No	9,938 (33.59)	7,908 (29.67)	9,347 (34.68)	589 (2.059)	<0.001
	Yes	22,745 (30)	20,752 (28.59)	28,267 (39.1)	1,694 (2.305)	
Exposure to mass media	No	9,379 (27.38)	9,383 (29.64)	12,949 (40.83)	686 (2.157)	<0.001
	Any	23,304 (32.68)	19,277 (28.55)	24,665 (36.5)	1,597 (2.272)	
Household and community factors						
Wealth index	Poorest	9,078 (27.06)	8,996 (29.45)	12,219 (41.43)	616 (2.07)	<0.001
	Poorer	7,880 (29.68)	6,999 (29.51)	9,168 (38.57)	540 (2.23)	
	Middle	6,290 (31.82)	5,391 (28.73)	6,986 (37.07)	485 (2.40)	
	Richer	5,363 (34.19)	4,192 (27.72)	5,458 (35.63)	395 (2.46)	
	Richest	4,072 (36.38)	3,082 (28.41)	3,783 (33.13)	247 (2.08)	
Household members	1 to 4	7,130 (32.46)	6,005 (29.48)	7,493 (36.03)	454 (2.04)	<0.001
	5 to 8	20,461 (30.67)	18,117 (28.84)	23,806 (38.14)	1,479 (2.35)	
	More than 8	5,092 (30.48)	4,538 (28.37)	6,315 (39.09)	350 (2.07)	
Social caste	Other	5,397 (32.61)	4,565 (28.92)	5,973 (36.34)	393 (2.13)	<0.001
	OBC	12,816 (32.96)	11,447 (28.81)	14,691 (36.25)	803 (1.98)	
	SC/ST	14,470 (27.71)	12,648 (28.98)	16,950 (40.69)	1,087 (2.62)	
Religion	Hindu	22,681 (30.62)	21,602 (28.87)	29,200 (38.27)	1,694 (2.24)	<0.001
	Muslims	3,782 (31.69)	3,507 (29.49)	4,788 (36.82)	335 (2.01)	
	Others	6,220 (36.27)	3,551 (27.32)	3,626 (33.53)	254 (2.87)	
Residents	Urban	6,680 (33.86)	5,323 (28.56)	6,585 (35.14)	462 (2.44)	<0.001
	Rural	26,003 (30.07)	23,337 (29.01)	31,029 (38.75)	1821 (2.2)	
Geographic region	East	6,024 (29.5)	6,682 (31.87)	7,829 (37.08)	287 (1.55)	<0.001
	West	2045 (25.09)	2,487 (28.72)	4,018 (43.34)	254 (2.85)	
	North	5,388 (29.42)	4,808 (28.08)	7,195 (39.27)	662 (3.23)	
	South	4,453 (37.84)	3,133 (25.86)	4,202 (33.88)	345 (2.42)	
	Central	8,262 (31.22)	7,629 (28.09)	10,787 (38.51)	602 (2.18)	
	Northeast	6,511 (38.29)	3,921 (31.07)	3,583 (29.91)	133 (0.73)	

Values are unweighted frequencies with survey-weighted row percentages in parentheses. *p*-values are from survey design-adjusted Rao–Scott tests. *p* < 0.05 was considered statistically significant.

The final adjusted multinomial logistic regression analysis Table 4 included 101,240 children after excluding observations with missing or invalid information on variables included in the adjusted model. The findings demonstrated that children born after an SBI had a significantly higher odds of moderate (aOR: 1.10; 95% CI: 1.05–1.15) and severe (aOR: 1.18; 95% CI: 1.05–1.34) anemia compared to those born after NSBI, with no significant association observed for mild anemia. Among child characteristics, age was strongly associated with anemia severity; children aged 42–59 months had a dramatically lower odds of moderate (aOR: 0.24; 95% CI: 0.23–0.26) and severe (aOR: 0.12; 95% CI: 0.10–0.14) anemia compared to those aged 6–23 months. Furthermore, birth weight <2,500 g was associated with a higher odd of moderate (aOR: 1.07; 95% CI: 1.00–1.13) and severe (aOR: 1.58; 95% CI: 1.38–1.82) anemia. Maternal health status was also strongly associated with childhood anemia severity, with maternal anemia showing a dose–response pattern; children of severely anemic mothers had a more than sevenfold higher odds of severe anemia (aOR: 7.04; 95% CI: 5.42–9.14) compared to children of non-anemic mothers. Additionally, maternal underweight status was associated with a higher odd of moderate (aOR: 1.13; 95% CI: 1.07–1.20) and severe (aOR: 1.23; 95% CI: 1.08–1.42) anemia.

**Table 4:** SBI and severity of childhood anemia in India: a multinomial logistic regression analysis.

Background characteristics		Mild anemia	Moderate anemia	Severe anemia
Birth interval	Non-short birth interval*®*	1 [1.00,1.00]	1 [1.00,1.00]	1 [1.00,1.00]
	Short birth interval	1.03 [0.98,1.08]	1.10*** [1.05,1.15]	1.18*** [1.05,1.34]
Child characteristics				
Child sex	Male*®*	1 [1.00,1.00]	1 [1.00,1.00]	1 [1.00,1.00]
	Female	0.99 [0.95,1.04]	0.97 [0.93,1.01]	0.85*** [0.76,0.96]
Child age (months)	6–23*®*	1 [1.00,1.00]	1 [1.00,1.00]	1 [1.00,1.00]
	24–41	0.78*** [0.74,0.83]	0.53*** [0.50,0.56]	0.46*** [0.40,0.52]
	42–59	0.56*** [0.53,0.60]	0.24*** [0.23,0.26]	0.12*** [0.10,0.14]
Birth weight	≥2,500 g *®*	1 [1.00,1.00]	1 [1.00,1.00]	1 [1.00,1.00]
	<2,500 g	0.96 [0.90,1.03]	1.07** [1.00,1.13]	1.58*** [1.38,1.82]
	Not weighed	1 [0.92,1.09]	1.06 [0.97,1.15]	1.53*** [1.21,1.92]
Birth order	Second birth *®*	1 [1.00,1.00]	1 [1.00,1.00]	1 [1.00,1.00]
	Third or higher birth	1.02 [0.97,1.08]	0.06* [0.01,1.12]	1.16** [1.00,1.33]
Maternal characteristics				
Women age (years)	15–24*®*	1 [1.00,1.00]	1 [1.00,1.00]	1 [1.00,1.00]
	25–34	0.96 [0.89,1.04]	0.86*** [0.80,0.92]	1.99 [0.82,1.20]
	> = 35	0.91** [0.82,1.02]	0.76*** [0.68,0.85]	0.89 [0.67,1.18]
Women education	No education*®*	1 [1.00,1.00]	1 [1.00,1.00]	1 [1.00,1.00]
	Primary	0.92** [0.85,0.99]	0.89*** [0.83,0.96]	0.81** [0.67,0.98]
	Secondary	0.89*** [0.84,0.95]	0.81*** [0.76,0.87]	0.71*** [0.61,0.84]
	Higher	0.83*** [0.76,0.92]	0.65*** [0.59,0.71]	0.52*** [0.40,0.67]
Age at first birth	Less than 20*®*	1 [1.00,1.00]	1 [1.00,1.00]	1 [1.00,1.00]
	20 to 29	1.02 [0.97,1.07]	1.05** [1.00,1.11]	0.99 [0.87,1.14]
	30 to 49	1.06 [0.86,1.31]	1.18 [0.97,1.43]	0.92 [0.57,1.47]
Place of delivery	Non-Institutional*®*	1 [1.00,1.00]	1 [1.00,1.00]	1 [1.00,1.00]
	Institutional	1.04 [0.96,1.12]	1.02 [0.95,1.10]	1.28** [1.04,1.58]
Maternal anemia	Not anemic*®*	1 [1.00,1.00]	1 [1.00,1.00]	1 [1.00,1.00]
	Mild	1.32*** [1.24,1.39]	1.55*** [1.47,1.64]	1.63*** [1.39,1.91]
	Moderate	1.58*** [1.50,1.67]	2.28*** [2.16,2.40]	2.93*** [2.54,3.38]
	Severe	1.42*** [1.21,1.68]	2.90*** [2.49,3.39]	7.04*** [5.42,9.14]
Maternal BMI	Normal*®*	1 [1.00,1.00]	1 [1.00,1.00]	1 [1.00,1.00]
	Underweight	1.04 [0.98,1.11]	1.13*** [1.07,1.20]	1.23*** [1.08,1.42]
	Overweight/Obese	0.93** [0.88,1.00]	0.89*** [0.84,0.95]	0.95 [0.80,1.12]
Received any benefits from the Anganwadi	No*®*	1 [1.00,1.00]	1 [1.00,1.00]	1 [1.00,1.00]
	Yes	1.03 [0.97,1.08]	1.08*** [1.02,1.13]	1.01 [0.88,1.16]
Exposure to mass media	No*®*	1 [1.00,1.00]	1 [1.00,1.00]	1 [1.00,1.00]
	Any	0.94** [0.89,0.99]	0.91*** [0.86,0.96]	0.97 [0.84,1.13]
Household and community factors				
Wealth index	Poorest*®*	1 [1.00,1.00]	1 [1.00,1.00]	1 [1.00,1.00]
	Poorer	1 [0.94,1.07]	0.93** [0.87,0.99]	1.02 [0.86,1.20]
	Middle	0.97 [0.90,1.05]	0.88*** [0.81,0.95]	1.01 [0.82,1.24]
	Richer	0.92* [0.84,1.00]	0.86*** [0.79,0.94]	1.04 [0.82,1.31]
	Richest	0.93 [0.83,1.04]	0.83*** [0.75,0.93]	0.88 [0.66,1.17]
Household members	1 to 4*®*	1 [1.00,1.00]	1 [1.00,1.00]	1 [1.00,1.00]
	5 to 8	0.99 [0.93,1.06]	1.06** [1.00,1.12]	1.11 [0.95,1.30]
	More than 8	0.97 [0.89,1.05]	1.05 [0.97,1.13]	0.93 [0.77,1.13]
Social caste	Other*®*	1 [1.00,1.00]	1 [1.00,1.00]	1 [1.00,1.00]
	OBC	1.01 [0.95,1.08]	0.98 [0.91,1.05]	0.93 [0.78,1.11]
	SC/ST	1.14*** [1.06,1.23]	1.18*** [1.10,1.27]	1.32*** [1.10,1.59]
Religion	Hindu*®*	1 [1.00,1.00]	1 [1.00,1.00]	1 [1.00,1.00]
	Muslims	1.03 [0.96,1.11]	1.01 [0.94,1.09]	0.95 [0.78,1.15]
	Others	0.82*** [0.73,0.91]	0.78*** [0.70,0.87]	1.1 [0.85,1.41]
Residents	Urban*®*	1 [1.00,1.00]	1 [1.00,1.00]	1 [1.00,1.00]
	Rural	1 [0.93,1.08]	1.01 [0.94,1.08]	0.86* [0.73,1.01]
Geographic region	East*®*	1 [1.00,1.00]	1 [1.00,1.00]	1 [1.00,1.00]
	West	1.24*** [1.12,1.38]	1.86*** [1.68,2.07]	2.88*** [2.25,3.69]
	North	1.03 [0.96,1.11]	1.43*** [1.32,1.55]	2.65*** [2.16,3.24]
	South	0.76*** [0.70,0.82]	1.03 [0.95,1.12]	1.77*** [1.40,2.23]
	Central	0.92*** [0.86,0.98]	1.18*** [1.11,1.27]	1.63*** [1.36,1.97]
	Northeast	0.84*** [0.77,0.92]	0.76*** [0.68,0.84]	0.41*** [0.28,0.59]
Number of observations	101,240			

AOR = Adjusted Odds Ratio; CI = Confidence Interval. Values in brackets represent 95% Cis; ® Reference category. And *p*-values are based on survey-weighted multinomial logistic regression accounting for the NFHS-5 complex survey design. Reference categories are denoted by ®. *p < 0.10, **p < 0.05, ***p < 0.01.

Socioeconomic and demographic factors were also associated with differences in childhood anemia severity. Higher maternal education was associated with a lower odd of severe anemia, with children of mothers with higher education having 48% lower odds of severe anemia (aOR: 0.52; 95% CI: 0.40–0.67) compared to those with no education. Social marginalization compounded vulnerability, as children from SC/ST communities had a significantly higher odds of moderate (aOR: 1.18; 95% CI: 1.10–1.27) and severe (aOR: 1.32; 95% CI: 1.10–1.59) anemia compared to the “Other” caste category. Geographic differences were also observed; compared with the East, children in the West (aOR: 2.88; 95% CI: 2.25–3.69), North (aOR: 2.65; 95% CI: 2.16–3.24), and Central (aOR: 1.63; 95% CI: 1.36–1.97) regions had a higher odd of severe anemia, whereas those in the Northeast had a lower odd (aOR: 0.41; 95% CI: 0.28–0.59). Finally, female children also had a lower odd of severe anemia than male children (aOR: 0.85; 95% CI: 0.76–0.96). Additionally, exposure to mass media was associated with a lower odd of moderate anemia (aOR: 0.91; 95% CI: 0.86–0.96).

## Discussion

This study examined the association between short birth intervals (SBI) and childhood anemia severity in India using secondary cross-sectional data from the NFHS-5. Applying survey-weighted multinomial logistic regression to 101,240 mother–child pairs, we found that children born after an SBI were significantly associated with a higher odd of moderate (aOR: 1.10) and severe anemia (aOR: 1.18) compared to those born after NSBI. Furthermore, a higher odd of severe anemia was independently associated with birth weight <2,500 g, severe maternal anemia, SC/ST caste, and residence in the West, North, or Central regions. Conversely, higher maternal education, greater household wealth, and residence in the Northeast were associated with a lower odd of severe anemia.

Although maternal nutritional depletion was not directly measured in this study, the observed associations are consistent with the possibility that maternal nutritional depletion may contribute to the observed relationship. This hypothesis posits that closely spaced pregnancies and concurrent lactation may prevent adequate replenishment of maternal micronutrient stores, particularly iron, folate, vitamins A and B12, zinc, and calcium (25). The female reproductive cycle comprises four phases: pregnancy, full lactation, partial lactation, and the non-pregnant non-lactating (NPNL) period. Conception during lactation may result in overlapping reproductive cycles that can increase maternal nutritional demands and, without adequate dietary and supplemental intake, may deplete maternal nutrient reserves (38, 40).

Biologically, severe maternal nutrient depletion may limit the transfer of iron to the fetus. This is associated with an increased odds of preterm birth and low birth weight, both of which leave the new born with critically low iron stores at birth (24, 41, 42). Postnatally, the physiological stress of breastfeeding-pregnancy overlap may degrade breast milk volume and immunological quality, potentially depriving the nursing infant of essential bioavailable iron (32). Behaviorally, concurrent childcare and pregnancy may lead to early weaning and the delayed introduction of nutrient-rich complementary foods (33). Moreover, closely spaced births may dilute household dietary, healthcare, and financial resources among multiple young children (23, 34). Together, these factors may create a cumulative risk environment that contributes to the severity of childhood anemia (35). These factors should be regarded as plausible contextual explanations rather than confirmed mechanisms.

Children aged 42–59 months had a lower odd of anemia compared with those aged 6–23 months, which may reflect greater dietary diversity and longer exposure to health interventions, including iron and folic acid (IFA) supplementation, deworming, and growth monitoring. The higher burden among children aged 6–23 months may be related to the critical transition from exclusive breastfeeding to complementary feeding, during which inadequate infant and young child feeding (IYCF) practices and nutritional deficiencies are more common (43–45). LBW infants had a higher odds of severe anemia, potentially reflecting lower iron stores at birth and higher iron requirements during rapid catch-up growth, which may increase their susceptibility to anemia (46). Preterm and growth-restricted infants may have reduced fetal iron accumulation, which may further increase their vulnerability to anemia (47). Immature immune function may increase susceptibility to infections, which may adversely affect nutrient absorption and iron status (45, 48). Additionally, LBW is associated with maternal undernutrition, maternal anemia, and SBI, and these interrelated factors may potentially contribute to the observed association with childhood anemia (49, 50).

Maternal anemia was significantly associated with childhood anemia. Although maternal anemia was measured at the time of the survey, maternal nutritional depletion may potentially contribute to this association, but should not be considered an established mechanism. Maternal anemia may also be associated with suboptimal breastfeeding practices and shared nutritional deficiencies, poverty, and exposure to infectious disease environments (30, 51, 52). Maternal undernutrition was also associated with childhood anemia, potentially through impaired placental growth and nutrient transfer, particularly iron, which may increase the likelihood of intrauterine growth restriction (IUGR), low birth weight, and lower neonatal iron stores (53). These disadvantages may be compounded by poverty, poor sanitation, and recurrent infections, potentially contributing to an intergenerational cycle of undernutrition and anemia (54, 55).

Maternal education was associated with lower odds of childhood anemia, potentially reflecting differences in health literacy, autonomy, and access to socioeconomic resources (56). More educated mothers may be better positioned to provide diverse, iron-rich diets, maintain better hygiene, and utilize preventive health services such as IFA supplementation and deworming (56). They also tend to delay childbearing and practice longer birth spacing, may be related to prevalence of low birth weight and low neonatal iron stores (57, 58). These factors may partly explain the observed association between maternal education and childhood anemia. Children from SC/ST households had a higher odds of childhood anemia, which may reflect of socioeconomic deprivation, poor living conditions, and intergenerational nutritional deprivation (59). Diets in some SC/ST communities may be predominantly cereal-based and contain fewer iron-rich foods, potentially limiting dietary iron intake and bioavailability (60). Geographic isolation and limited access to sanitation, healthcare, and nutrition services may further contribute to the observed disparities in childhood anemia (61, 62). Additionally, higher levels of maternal anemia, early marriage, and (SBI) may also be associated with adverse maternal and child nutritional conditions, including low birth weight and reduced neonatal iron stores, predisposing children to severe anemia from early life (37, 63).

The higher odds of childhood anemia in North India may be associated with entrenched patriarchal norms, limited dietary diversity, cereal-based diets with lower consumption of animal-source foods, and poor sanitation and infectious disease burden (60, 64). In Central India, geographic isolation, a higher concentration of SC/ST populations, and endemic malaria and hemoglobinopathies may contribute to the observed association (65). In contrast, the Northeast showed a lower odd potentially reflecting greater female autonomy, lower fertility, and more diverse diets rich in animal-source foods. Regional and caste disparities suggest the influence of broader structural and nutritional inequalities (66, 67). SBI were associated with childhood anemia, while maternal anemia, undernutrition, low birth weight, poverty, and social disadvantage may further contribute to vulnerability. These findings support region-specific strategies addressing maternal nutrition, birth spacing, IYCF practices, and socially and geographically marginalized populations. Given the cross-sectional design, these associations do not imply causality.

## Conclusion

This study found that children born after an SBI had a higher odd of moderate and severe anemia compared with those born after NSBI. Maternal nutritional depletion may be one possible explanation for this observed association, as closely spaced pregnancies and overlapping lactation periods may reduce maternal micronutrient stores, particularly iron, potentially affecting fetal iron accretion and infant nutritional status. Beyond birth spacing the burden was disproportionately higher among children aged 6–23 months, birth weight <2,500 g infants, and children of anemic or undernourished mothers. Marked socioeconomic and geographic inequalities were also evident, with children from SC/ST communities, and the North, West, Central and South regions experiencing substantially higher odds of severe anemia. In contrast, higher maternal education, greater household wealth, and residence in the Northeast were associated with a lower odd of childhood anemia. These findings highlight the need for geographically tailored and life-course-specific interventions that integrate family planning, promotion of optimal birth spacing, maternal nutrition, and improved infant and young child feeding practices. Targeted interventions for socially and geographically marginalized populations are particularly essential. However, given the cross-sectional nature of NFHS-5, the observed associations should be interpreted cautiously, and causal relationships cannot be established. Longitudinal studies are needed to better understand the causal pathways linking birth spacing and childhood anemia. Nevertheless, addressing these interconnected factors may help reduce the burden of childhood anemia and support progress toward the SDGs on zero hunger and good health and well-being.

## Strengths and limitations

This study possesses several notable strengths. First, it utilizes a large, nationally representative sample from the NFHS-5, ensuring high generalizability across India’s diverse demographic landscape. Second, the methodological rigor is robust; by employing survey-weighted multinomial logistic regression, the analysis appropriately accounts for the complex survey design and examines graded anemia severity (mild, moderate, and severe) rather than a simplistic binary outcome. Third, the comprehensive adjustment for child, maternal, household, and geographic covariates minimize confounding bias, providing a nuanced understanding of intersecting structural inequities.

However, certain limitations must be acknowledged. Primarily, the cross-sectional design precludes the establishment of causal relationships between short birth intervals and childhood anemia; longitudinal studies are required to confirm these pathways. Second, the restriction to ever-married women limit the generalizability of the findings to children born to never-married mothers, though this group is demographically small in India. Third, while the maternal nutritional depletion hypothesis provides a plausible biological explanation, it was not directly measured in this study, meaning these mechanisms remain inferential rather than empirically confirmed. Finally, we employed complete-case analysis because 75.17% of missing data occurred in primary exposure or outcome variables due to structural non-response, where imputation risks introducing artificial bias. Although this approach is standard, it may introduce selection bias if missingness is not entirely random. Consequently, findings may have limited generalizability to highly mobile or non-responding subpopulations. Despite these constraints, the findings offer critical, actionable insights for public health interventions targeting vulnerable populations across India.

## Data Availability

Publicly available datasets were analyzed in this study. This data can be found at: https://www.dhsprogram.com/data/.
